# The role of ephaptic coupling and gap junctional coupling in modulating the initiation and dynamics of reentrant arrhythmias

**DOI:** 10.1371/journal.pone.0330016

**Published:** 2025-08-19

**Authors:** Ning Wei, Elena G. Tolkacheva

**Affiliations:** 1 Department of Mathematics, Purdue University, 150 N. University St, West Lafayette, Indiana, United States of America; 2 Department of Biomedical Engineering, University of Minnesota, 312 Church St SE, Minneapolis, Minnesota, United States of America; Campus Bio-Medico University Departmental Faculty of Medicine and Surgery: Universita Campus Bio-Medico di Roma Facolta Dipartimentale di Medicina e Chirurgia, ITALY

## Abstract

Cardiac myocytes synchronize through electrical signaling to contract heart muscles, facilitated by gap junctions (GJs) located in the intercalated disc (ID). GJs provide low-resistance pathways for electrical impulse propagation between myocytes, considered the primary mechanism for electrical communication in the heart. However, research indicates that conduction can persist without GJs. Ephaptic coupling (EpC), which depends on electrical fields in the narrow ID between adjacent myocytes, offers an alternative mechanism for cardiac conduction when GJs are impaired. Research suggests that EpC can enhance conduction velocity (CV) and reduce the likelihood of conduction block (CB), particularly when GJs are impaired, demonstrating the anti-arrhythmic potential of EpC. Reduced GJ communication increases the susceptibility of heart to arrhythmias due to ectopic or triggered activity, highlighting the pro-arrhythmic effect of GJ uncoupling. However, the interplay between GJs and EpC, and their roles in the initiation, dynamics, and termination of arrhythmias, remain unclear. Reentry, characterized by a loop of electrical activity, is a common mechanism underlying arrhythmogenesis in the heart. This study aims to explore the interplay between EpC and GJs on reentry initiation and its underlying dynamics. Specifically, we employed a two-dimensional (2D) discrete bidomain model that integrates EpC to simulate ephaptic conduction during reentry. We quantitatively assessed the outcomes of reentry initiation and the resulting dynamics across different levels of EpC, GJs, and initial perturbations. The results show that sufficiently strong EpC (i.e., sufficiently narrow clefts) tends to suppress reentry initiation, resulting in absent or non-sustained reentrant activity, while also introducing transient instability and heterogeneity into the cardiac dynamics. In contrast, relatively weak EpC (wide clefts) support sustained reentry with a stable rotor. Furthermore, we found that sufficiently strong EpC can lower the maximal dominant frequency observed during reentrant activity. Overall, this suggests that strong EpC exerts an anti-arrhythmic effect.

## Introduction

Cardiac myocytes synchronize to contract the heart muscles, facilitating blood circulation through electrical signaling. Gap junctions (GJs), located in the intercalated disc (ID) act as low-resistance pathways facilitating electrical connections between cardiac myocytes, thereby enabling the propagation of electrical impulses [2–5]. GJ is widely recognized as the primary mechanism for electrical communication between myocytes [6]. However, recent experimental findings have raised concerns regarding whether conduction in the heart can be sustained without GJs [7, 8]. For example, mice with GJ knockout [8] still exhibit electrical propagation in the heart, albeit with discontinuous and slow conduction. Remarkably, these mice can survive for up to two months, suggesting the existence of alternative mechanisms for cell-to-cell communication in the heart.

Ephaptic coupling (EpC) serves as an alternative mechanism for cardiac conduction when GJs are dysfunctional. Studies have shown that EpC relies heavily on the electrical fields within the narrow clefts between neighboring myocytes [9, 10]. Since the middle of 20th century, EpC has undergone extensive experimental and numerical investigations, yet direct experimental evidence for its existence remains elusive. Consequently, efforts have been made to indirectly demonstrate the presence of EpC by studying its physiological role in the heart under various conditions [1, 9, 11–21]. For example, research indicates that EpC can assist in restoring cardiac conduction when GJs are impaired by enhancing conduction velocity (CV) [12, 13, 16, 21–23] and mitigating conduction block (CB) [21].

Reduced gap junctional communication can make the heart more susceptible to ectopic or triggered activity from afterdepolarizations, thus contributing to arrhythmias [6, 24, 25]. GJs are significantly reduced during myocardial ischemia, a condition characterized by reduced blood flow to the heart muscle, resulting in insufficient oxygen supply. Ischemic conditions disrupt normal electrical activity of the heart, increasing the likelihood of arrhythmias. We demonstrated that sufficient strong EpC (narrow cleft) can significantly diminish CB across the ischemic tissue [15], suggesting a beneficial effect of EpC on arrhythmogenesis in the ischemic heart. We also demonstrated that sufficiently strong EpC terminates reentry in healthy and ischemic heart [1]. Reentry is a primary mechanism underlying arrhythmogenesis in the heart, including atrial fibrillation and ventricular tachycardia. Despite these advancements, the interplay between EpC and GJs on the initiation, dynamics, and termination of cardiac arrhythmias remains elusive.

The electrophysiological properties of cardiac tissue, such as CV, refractoriness, and excitability, are key factors influencing arrhythmic dynamics, particularly the spatio-temporal behavior of electrical activity in the heart. Understanding reentry initiation and its underlying dynamics is essential for improving the treatment and management of cardiac arrhythmias. The goal of this study is to explore how different levels of EpC and GJs influence the initiation and dynamics of reentry, utilizing a two-dimensional (2D) discrete bidomain model of cardiac conduction incorporating EpC. Specifically, we quantitatively assessed the incidence of reentry initiation and analyzed the subsequent dynamics across different levels of EpC, GJs, and initial perturbations. The results show that sufficiently strong EpC (i.e., sufficiently narrow clefts) tends to suppress reentry initiation, resulting in absent or non-sustained reentrant activity, while also introducing transient instability and heterogeneity into the cardiac dynamics. In contrast, relatively weak EpC (wide clefts) supports sustained reentry with a stable rotor. Furthermore, we found that sufficiently strong EpC can lower the maximal dominant frequency (maxDF) observed during reentrant activity. Overall, this suggests that strong EpC exerts an anti-arrhythmic effect.

## Materials and methods

### 2D model overview

We employed our previously developed 2D discrete bidomain model with EpC [1, 15, 21] to simulate cardiac conduction, which is strongly influenced by the junctional cleft space between neighboring cells. In this model, each cell is represented as a cylinder, and the cells are interconnected via Gjs to form an M×N rectangular lattice. At each lattice point (*i*, *j*), both the intracellular potential, $\phi_{i}^{\left(i,j\right)}$, and the extracellular potential, $\phi_{e}^{\left(i,j\right)}$, are defined. The junctional cleft is positioned between adjacent cells (*i*, *j*) and (i,j  +  1), and we introduced a cleft potential, $\phi_{c}^{\left(i,j+\frac{1}{2}\right)}$, at the location (i,j  +  $\frac{1}{2})$. The space adjacent to the cleft, which lies within the extracellular region, is called the extracellular-cleft space, with its potential denoted as $\phi_{ec}^{\left(i,j+\frac{1}{2}\right)}$.

The top panel of Fig 1 in [21] shows the lattice view of the model, while the bottom panel provides a circuit diagram representing two adjacent cells connected through the shared cleft space and end-to-end gap junctions ($GJ_{\text{end}}$). Note that side-to-side gap junctions ($GJ_{\text{side}}$) and the resistive connection (*R*_*ee*_) between extracellular spaces in the transverse direction are not included. The cleft space is modeled as a narrow compartment with resistive connections (*R*_*c*_) to the extracellular-cleft space, and the resistive connections between the extracellular and extracellular-cleft spaces are represented by *R*_*ec*_. The intracellular and extracellular spaces of each cell are separated by the side membrane, while the intracellular and cleft spaces are separated by the end membrane. These side and end membranes function independently, allowing for the flow of ionic and capacitive currents. To simplify the computations, we assume that the intracellular and extracellular spaces of each cell are isopotential.

**Fig 1:**
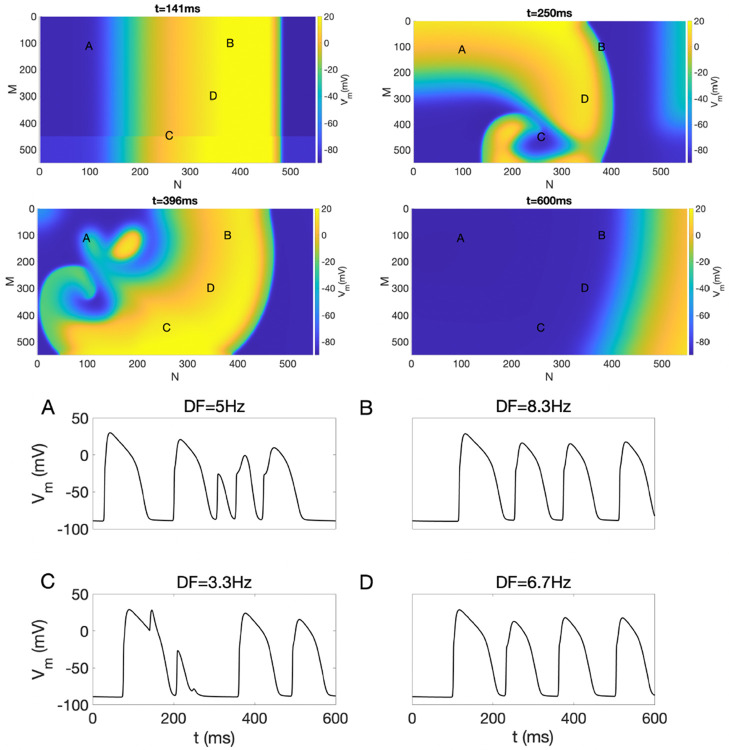
Top: Snapshots of nonsustained reentry in the presence of EpC at various time points (t) with $d_{cleft}=15$ nm and 100% GJ. Bottom: Representative $V_{m}$ traces for points A, B, C, and D, along with their corresponding DF values. The color bar shows the $V_{m}$ values.

### Modeling EpC

EpC is critically dependent on the presence of a cleft space between the ends of adjacent cells, which communicates with the end membranes of neighboring cells and the extracellular space independently. To model EpC, we derived an equation for the cleft space based on the principle of current conservation. This equation incorporates the balance of capacitive and ionic currents across the end membranes of two adjacent cells sharing a common cleft potential, as well as the resistive current flowing from the cleft into the extracellular space, characterized by *R*_*c*_. The current balance is captured by the following expression:

$$-A_{\text{end}}C_{m}\frac{\partial (\phi_{i}^{\left(i,j\right)}-\phi_{\text{c}}^{\left(i,j+\frac{1}{2}\right)})}{\partial t}-A_{\text{end}}C_{m}\frac{\partial (\phi_{i}^{\left(i,j+1\right)}-\phi_{\text{c}}^{\left(i,j+\frac{1}{2}\right)})}{\partial t}-I_{\text{end}}\;+\frac{\phi_{c}^{\left(i,j+\frac{1}{2}\right)}-\phi_{ec}^{\left(i,j+\frac{1}{2}\right)}}{R_{c}}=0.$$ (1)

*R*_*c*_ is inversely proportional to the cleft width ($d_{\text{cleft}}$), with the relevant formulas available in [21, 23]. We chose $d_{\text{cleft}}$ values of 8 nm, 15 nm, 20 nm, and 35 nm [1, 15, 21] to represent varying strengths of EpC. Notably, a smaller $d_{\text{cleft}}$ corresponds to higher *R*_*c*_, indicating a stronger EpC effect. A $d_{\text{cleft}}$ of 115 nm indicates a negligible EpC effect.

The model equations were derived using current balance principles for the intracellular, extracellular, cleft, and extracellular-cleft spaces. Specifically, the current balance captures the equilibrium among capacitive, ionic, and resistive currents across the different domains. The detailed equations are presented in Eqs (2.1) to (2.4) of our previous publication [21], and all model parameters are provided in Table 1 of Ref. [21].

**Table 1:** Classification of reentry initiation: Outcomes are categorized as sustained, non-sustained, or no reentry across varying levels of GJs, EpC, and initial condition perturbations.

GJ	Perturbation of initial condition	EpC (represented by $d_{cleft}$ in nm)				
		8 nm	15 nm	20 nm	35 nm	115 nm
100%	-20%	Nonsustained	Nonsustained	Nonsustained	Sustained	Sustained
	–10%	Nonsustained	Nonsustained	Nonsustained	Sustained	Sustained
	0%	No	Nonsustained	Nonsustained	Sustained	Sustained
	+10%	No	Nonsustained	Sustained	Sustained	Sustained
	+20%	No	Nonsustained	Nonsustained	Sustained	Sustained
80%	-20%	Nonsustained	Nonsustained	Nonsustained	Sustained	Sustained
	–10%	Nonsustained	Nonsustained	Sustained	Sustained	Sustained
	0%	No	Nonsustained	Nonsustained	Sustained	Sustained
	+10%	No	Nonsustained	Sustained	Nonsustained	Sustained
	+20%	No	Nonsustained	Nonsustained	Sustained	Sustained
50%	-20%	Nonsustained	Nonsustained	Nonsustained	Sustained	Sustained
	–10%	Nonsustained	Sustained	Sustained	Sustained	Sustained
	0%	No	Nonsustained	Nonsustained	Nonsustained	Sustained
	+10%	No	Nonsustained	Sustained	Sustained	Sustained
	+20%	No	Nonsustained	Nonsustained	Sustained	Sustained
30%	-20%	Nonsustained	Nonsustained	Sustained	Sustained	Sustained
	–10%	Nonsustained	Nonsustained	Nonsustained	Sustained	Sustained
	0%	Nonsustained	Nonsustained	Sustained	Nonsustained	Sustained
	+10%	No	Nonsustained	Sustained	Sustained	Sustained
	+20%	No	Nonsustained	Nonsustained	Sustained	Sustained
10%	-20%	Sustained	Nonsustained	Sustained	Sustained	Sustained
	–10%	Nonsustained	Nonsustained	Nonsustained	Sustained	Sustained
	0%	Nonsustained	Nonsustained	Sustained	Sustained	Sustained
	+10%	No	Sustained	Sustained	Sustained	Sustained
	+20%	No	Nonsustained	Sustained	Sustained	Sustained

### Membrane dynamics

To model the dynamics of excitable cells in normal tissue, we used the Luo-Rudy dynamic model 2007 (LRd2007), which is specific to guinea pig ventricular tissue [26]. In our 2D model, we localized the fast sodium (INa) channels to the end membrane, as observed in experimental studies [27–29]. This localization was achieved by redistributing the INa channels across the end membrane, while maintaining a constant total number of ionic channels or conductance. Other ionic channels are uniformly distributed across both the side and end membranes, with the channels and their gating variables functioning independently on each membrane.

### Analysis of reentry dynamics

Our goal is to thoroughly examine the effects of EpC and GJs on reentry initiation and the resulting dynamics. To accomplish this, we employed different levels of GJs at 100%, 80%, 50%, 30%, and 10% of the nominal value (666 mS/cm^2^ [21]), along with varying levels of EpC. Additionally, for each combination of GJ and EpC, we introduced ±10% and ±20% perturbations from the steady state to the initial conditions of the potentials, ionic concentrations, and gating variables of all ionic currents.

We quantitatively evaluated reentry initiation outcomes, categorizing them as sustained reentry, nonsustained reentry, or no reentry. Additionally, we calculated the maxDF and the number of regions in the 2D DF maps (#DF). In the context of arrhythmia, DF represents the frequency of the highest peak in the power spectrum of cardiac electrical activity, reflecting the primary frequency of reentrant circuits or other rapid drivers in the heart. DF is calculated by applying the fast Fourier transform (FFT) to the potential signals generated by our 2D model. It is widely used to characterize arrhythmia dynamics [30–32] and plays a crucial role in assessing the pro- or anti-arrhythmic effects of EpC. The maxDF corresponds to the highest frequency detected at a specific site in the time series, identifying regions with the most rapid reentrant or focal sources. The #DF reflects the spatial complexity of reentry, with a higher count suggesting more fragmented and potentially unstable reentrant pathways. Together, these metrics offer a comprehensive evaluation of the stability, speed, and spatial organization of reentrant arrhythmias under varying conditions.

### Numerical simulations

Numerical simulations were performed on a *M* × *N* lattice (*M* = *N* =  550) with a total simulation duration of 1200 ms or more. Each cell had a length of 0.01 cm and a radius of 0.0011 cm, with a time step of 0.01 ms. To initiate reentry, we applied an S1-S2 cross-field stimulation protocol. The initial stimulus (S1) was delivered to the left boundary of the lattice at time (t)=0, with an amplitude of 0.15 μA and a duration of 2 ms. Following this, the second stimulus (S2) was administered to the bottom of the lattice at the S1-S2 interval, while preserving the same amplitude and duration. To minimize the impact of the pacing protocol on reentry dynamics, we maintained a consistent S1-S2 interval (140 ms) across different levels of EpC, GJ and initial perturbations.

To solve the system, we used a splitting method, which enabled us to update the potential, ionic concentrations, and gating variables of ion channels independently. Specifically, we handled the linear components of the system using the backward Euler method, while linearizing the nonlinear components (such as ionic currents and dynamics) around the values from the previous time step, and then managing them with the same method. The system was solved using a direct method, specifically the backslash operator in Matlab. The wavefront of typical action potential propagation is identified as the point in space where the lateral transmembrane potential ($V_{m}$) exceeds -30 mV, accompanied by a positive temporal derivative ($\frac{\partial V_{m}}{\partial t}>0$). Longitudinal conduction was monitored by determining the earliest activation time (EAT) at each column of the M×N lattice, with activation initiated from the left side. Longitudinal CV (CV_*L*_) was calculated using linear regression of the EAT across 20–80% of the lattice length to minimize boundary effects. Transverse CV (CV_*T*_) was calculated in a similar manner by initiating activation from the bottom of the lattice. The anisotropy ratio was defined as the ratio of CV_*L*_ to CV_*T*_. The refractory period was estimated using the longest S1–S2 interval at which longitudinal conduction of the S2 beat fails—serving as a reliable indicator of tissue refractoriness.

## Results

### The impact of EpC on reentry dynamics

**Fig 2:**
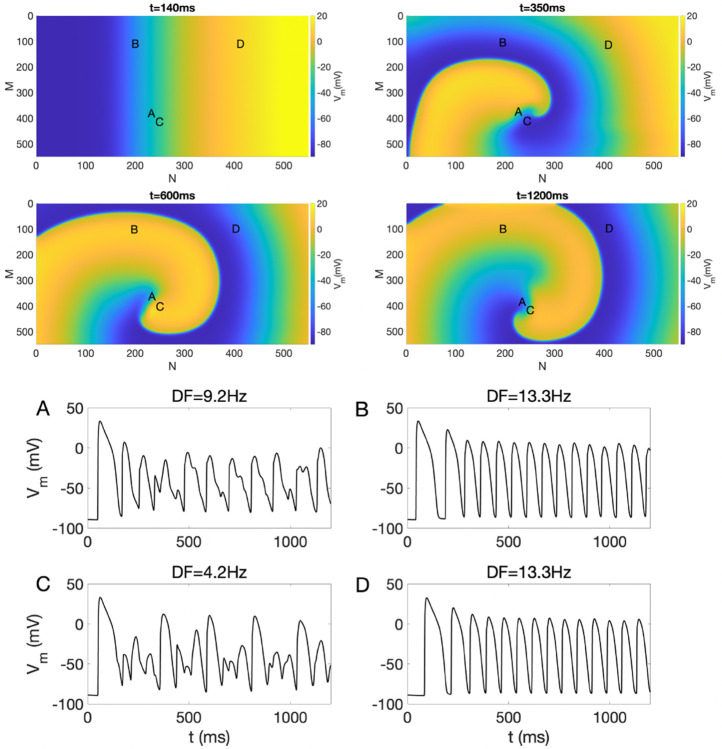
Top: Snapshots of sustained reentry in the near absence of EpC at various time points (t) with $d_{cleft}=115$ nm and 100% GJ. Bottom: Representative $V_{m}$ traces for points A, B, C, and D, along with their corresponding DF values. The color bar shows the $V_{m}$ values.

We first induced reentry in our 2D model using the cross-field pacing protocol with an S1-S2 interval of 140 ms, with steady-state as the initial setup. The values of $d_{\text{cleft}}$ were set to 15 nm and 115 nm, with GJs kept at 100%. Top panels of Figs 1 and 2 present snapshots of $V_{m}$ at different time points, illustrating nonsustained and sustained reentry in the presence ($d_{\text{cleft}}=15$ nm) and absence ($d_{\text{cleft}}=115$ nm) of EpC, respectively. The bottom panels of both figures display representative $V_{m}$ traces at points A, B, C and D along with the corresponding DF values.

As illustrated in the top panel of Fig 1, the reentry dissipates over time and is not sustained in the presence of EpC. Conversely, the top panel of Fig 2 demonstrates a stable and persistent reentrant pattern in the near absence of EpC. As indicated in the bottom panel of both figures, the $V_{m}$ traces for points A and C, located at the center of the reentry, exhibit chaotic behavior, while the $V_{m}$ traces for points B and D, situated at the periphery of the reentry, display a rapid yet regular pattern. It’s important to note that the DFs calculated from potential traces at points A and C are lower compared to those at points B and D. The center of reentry has a smaller DF compared to the periphery due to the nature of the electrical activity in these regions. At the center of the reentrant circuit, the wavefront is constantly turning, leading to more complex and slower conduction patterns, which reduces the DF. In contrast, the periphery experiences faster and more regular conduction, resulting in a higher DF. This difference is driven by the dynamics of wavefront propagation and the curvature of the reentrant pathway, which is more pronounced at the center. Comparing Fig 1 with Fig 2, one can suggest that EpC can reduce DF. Consequently, with strong EpC, DF may not reach the level needed for sustained reentry, indicating that EpC can suppress the initiation of reentry.

**Fig 3:**
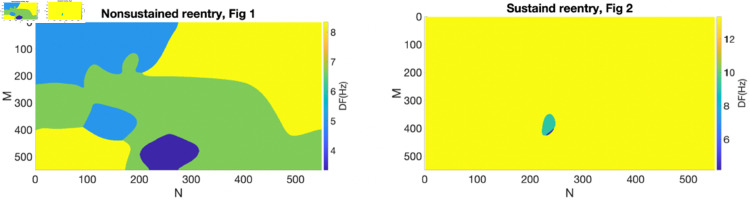
The 2D DF maps for nonsustained and sustained reentry shown in Figs 1 and 2, respectively, at an S1-S2 interval of 140 ms.

Fig 3 presents 2D DF maps for Figs 1 and 2, where reentry is nonsustained and sustained, respectively. It is evident that the maxDF is considerably lower for nonsustained reentry (8 Hz, left) compared to sustained reentry (13 Hz, right). However, the #DF is greater for nonsustained reentry (6 regions, left) than for sustained reentry (3 regions, right).

**Fig 4:**
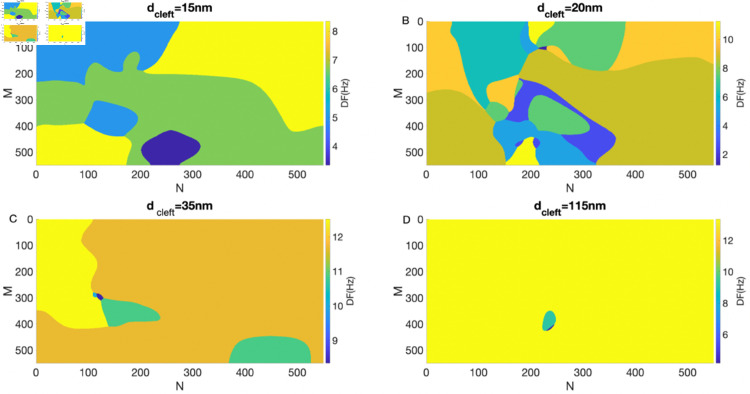
2D DF maps for various $d_{cleft}$ values (15 nm, 20 nm, 35 nm, and 115 nm in panels A–D) with 100% GJ. The colorbar indicates DF values (in Hz) derived from reentry at an S1-S2 interval of 140 ms.

Fig 4 presents the 2D DF maps for $d_{\text{cleft}}=15$ nm, 20 nm, 35 nm, and 115 nm with 100% GJ coupling. Reentry did not occur at $d_{\text{cleft}}=8$ nm; therefore, its corresponding DF map is not shown. Notably, reentry is nonsustained for $d_{\text{cleft}}=15$ nm and 20 nm, whereas sustained reentry is observed at 35 nm and 115 nm. This suggests that stronger EpC (i.e., smaller $d_{\text{cleft}}$) suppresses the initiation of reentry. To further understand the impact of EpC on the dynamics of reentry, we examined the maxDF and the #DF across the 2D lattice. The results indicate that the maxDF increases with larger $d_{\text{cleft}}$, while the #DF shows a biphasic trend—first increasing and then decreasing with $d_{\text{cleft}}$—with the maximum #DF (max#DF) observed at $d_{\text{cleft}}=20$ nm. These findings suggest that EpC reduces the maxDF and exerts a dual influence on the spatial complexity of reentry.

### Impact of EpC and GJs on reentry initiation and subsequent dynamics

To thoroughly investigate the effects of EpC and GJs on reentry initiation and the resulting dynamics, we assessed outcomes across a range of EpC levels, GJs, and initial perturbations. As summarized in Table 1, reentry initiation was classified as sustained, nonsustained, or absent. The results indicate that smaller $d_{\text{cleft}}$ values are more likely to result in nonsustained or no reentry, while wider clefts tend to promote sustained reentry, regardless of the level of GJs and initial perturbations. In particular, at $d_{\text{cleft}}=8$nm, nearly all cases result in either nonsustained reentry or no reentry. In contrast, sustained reentry consistently occurs when EpC was minimal ($d_{\text{cleft}}=115$ nm). Additionally, reduced GJs increased the probability of both nonsustained and sustained reentry across all $d_{\text{cleft}}$ values. The results above demonstrate that strong EpC suppresses reentry initiation, indicating an anti-arrhythmic effect.

**Fig 5:**
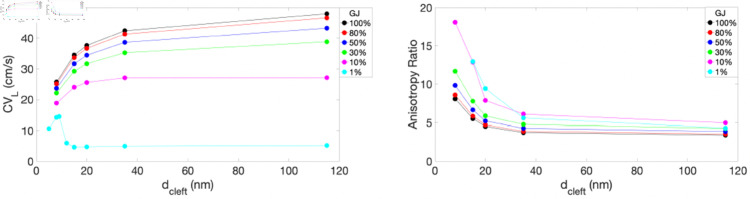
CV_*L*_ (left) and anisotropy ratio (right) as a function of $d_{cleft}$, shown for various levels of GJs: 100% (black), 80% (red), 50% (blue), 30% (green), 10% (magenta) and 1% (cyan).

We next examined cardiac dynamics both prior to and following the onset of reentry. Specifically, we investigated how EpC and GJ coupling influence CV_*L*_ (left) and the anisotropy ratio (${\text{CV}}_{\text{L}}/{\text{CV}}_{\text{T}}$, right) before reentry develops, as illustrated in Fig 5. As shown in the left panel, CV_*L*_ decreases with stronger EpC (i.e., as $d_{\text{cleft}}$ decreases) when GJ is equal to or greater than 10%. However, when GJ is sufficiently low (e.g., 1%), CV_*L*_ exhibits a biphasic relationship with $d_{\text{cleft}}$, consistent with findings reported in previous studies [21, 23, 33, 34]. In this case, an optimal $d_{\text{cleft}}$ of 9 nm yields the maximum CV_*L*_, reaching approximately 15 cm/s. In the right panel, the anisotropy ratio consistently increases as the cleft narrows across all levels of GJ, indicating that conduction becomes more strongly biased in the longitudinal direction when EpC is enhanced. Moreover, this directional bias becomes even more pronounced when GJ is reduced.

**Fig 6:**
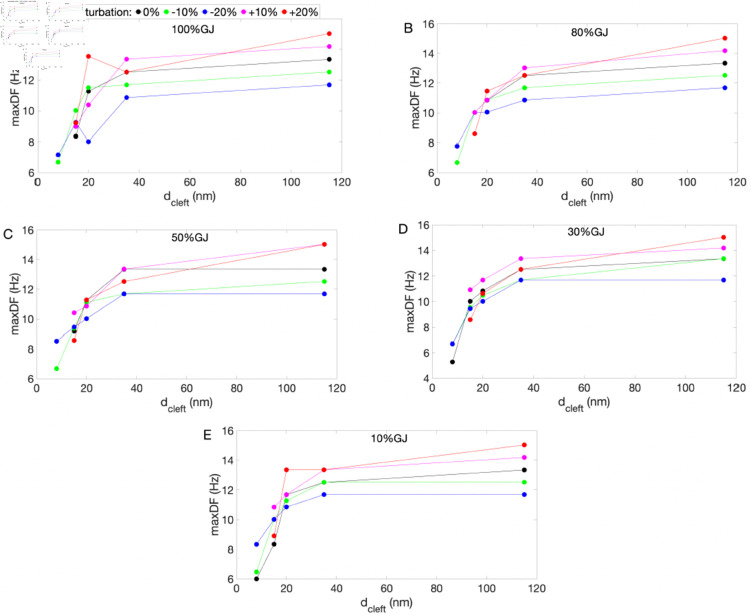
maxDF across varying levels of EpC (represented by $d_{cleft}$), GJs–100% (A), 80% (B), 50% (C), 30% (D), and 10% (E)–and initial condition perturbations: 0% (black), –10% (green), –20% (blue), +10% (magenta), and +20% (red). Results correspond to both nonsustained and sustained reentry summarized in Table 1.

**Fig 7:**
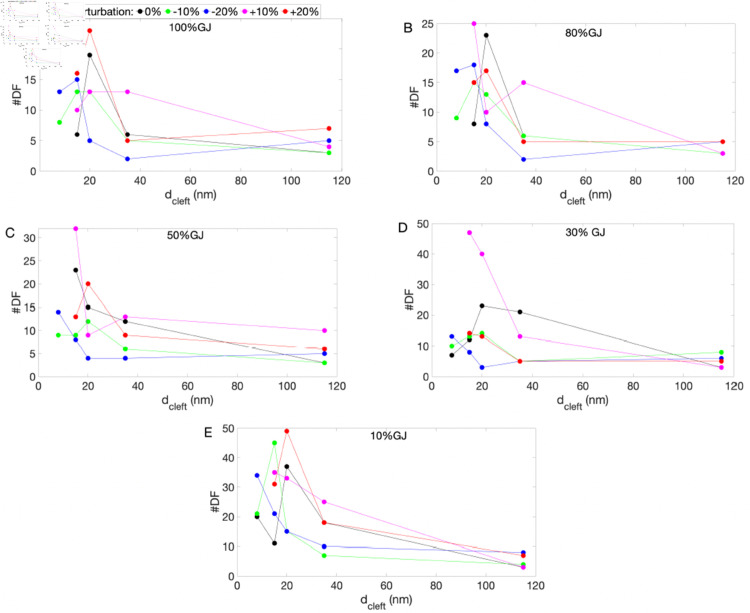
The #DF across varying levels of EpC (represented by $d_{cleft}$), GJs–100% (A), 80% (B), 50% (C), 30% (D), and 10% (E)–and initial condition perturbations: 0% (black), –10% (green), –20% (blue), +10% (magenta), and +20% (red). Results correspond to both nonsustained and sustained reentry summarized in Table 1.

Additionally, we analyzed the maxDF (Fig 6) and #DF (Fig 7) across different levels of EpC, GJ, and initial perturbations for both nonsustained and sustained reentry, as outlined in Table 1. As shown in Fig 6, maxDF decreases as $d_{\text{cleft}}$ decreases. While there are slight variations in the trend depending on the initial perturbations and levels of GJs, the overall trend remains consistent. This suggests that EpC has the potential to lower the maxDF, indicating an anti-arrhythmic effect of EpC.

Table 2 summarizes the $d_{\text{cleft}}$ values at which the #DF is maximized, across different GJ levels and initial condition perturbations. Italicized and bolded entries correspond to sustained and non-sustained reentry, respectively. Fig 7 and Table 2 shows that the #DF exhibits a nonlinear response to variations in $d_{\text{cleft}}$, with max#DF typically occurring at $d_{\text{cleft}}=15$ nm or 20 nm—indicative of moderate EpC strength. Additionally, there are instances where the #DF is maximized at $d_{\text{cleft}}=8$ nm, corresponding to either nonsustained or sustained reentry. Table 2 shows that, although the max#DF occur at varying $d_{\text{cleft}}$ values, they are predominantly associated with nonsustained reentry. In summary, a sufficiently narrow cleft may prevent the initiation of reentry but can also cause the reentrant wavefront to meander through the tissue. This leads to spatial and temporal irregularities in activation patterns, resulting from wavebreaks, conduction heterogeneities, or multiple competing sources. However, these instabilities and heterogeneities in cardiac dynamics are transient and tend to dissipate over time. In contrast, at wider cleft widths, reentry is sustained, and the rotor remains stable. This suggests that while sufficiently strong EpC may suppress the initiation of reentry, it can also temporarily promote more fragmented electrical activity.

**Table 2:** Identification of specific $d_{\text{cleft}}$ values at which the #DF is maximized across varying levels of GJ coupling and initial condition perturbations. Italicized and bolded entries correspond to sustained and non-sustained reentry, respectively.

GJ	Perturbation of initial condition	EpC (represented by $d_{cleft}$ in nm)				
		8 nm	15 nm	20 nm	35 nm	115 nm
100%	-20%		**max#DF**			
	–10%		**max#DF**			
	0%			**max#DF**		
	+10%			*max#DF*		
	+20%			**max#DF**		
80%	-20%		**max#DF**			
	–10%		**max#DF**			
	0%			**max#DF**		
	+10%		**max#DF**			
	+20%			**max#DF**		
50%	-20%	**max#DF**				
	–10%		*max#DF*			
	0%		**max#DF**			
	+10%		**max#DF**			
	+20%			**max#DF**		
30%	-20%	**max#DF**				
	–10%			**max#DF**		
	0%			*max#DF*		
	+10%		**max#DF**			
	+20%		**max#DF**			
10%	-20%	*max#DF*				
	–10%		**max#DF**			
	0%			*max#DF*		
	+10%		*max#DF*			
	+20%			*max#DF*		

## Discussion

The role of EpC in cardiac arrhythmias is of considerable scientific and clinical interest. Investigating its effects is crucial for advancing our understanding of arrhythmogenesis. In our previous study [1], we demonstrated that EpC can terminate reentrant activity under both normal and ischemic conditions. However, the precise mechanisms underlying this termination—and the broader influence of EpC on the initiation and dynamics of reentry—remain incompletely understood. In the present work, we focused on analyzing how EpC affects reentry initiation and subsequent dynamics, using DF and CV analysis as primary tools. DF, maxDF, and #DF serve as quantitative measures to evaluate the frequency distribution and spatial organization of cardiac electrical activity. DF represents the predominant frequency of activation in the tissue. Higher DF typically reflects faster activation rates, often driven by stable reentrant circuits or high-frequency rotors. This can lead to organized but rapid arrhythmias, such as atrial flutter or certain forms of ventricular tachycardia. In such cases, instability may arise from the tissue’s inability to follow high-frequency pacing, which can result in conduction block or degeneration into fibrillation [35–38]. Fragmented DF, on the other hand, suggests spatial or temporal irregularity in activation patterns. This is often associated with wavebreaks, conduction heterogeneities, or multiple competing sources, which are hallmarks of fibrillatory conduction. Such fragmentation reflects a loss of synchrony and is commonly observed in disorganized arrhythmias like atrial or ventricular fibrillation [39, 40]. Analyzing these DF-based parameters provides critical insights into the mechanisms of arrhythmia initiation and maintenance, which may guide targeted therapeutic strategies and improve risk stratification in clinical settings.

**Fig 8:**
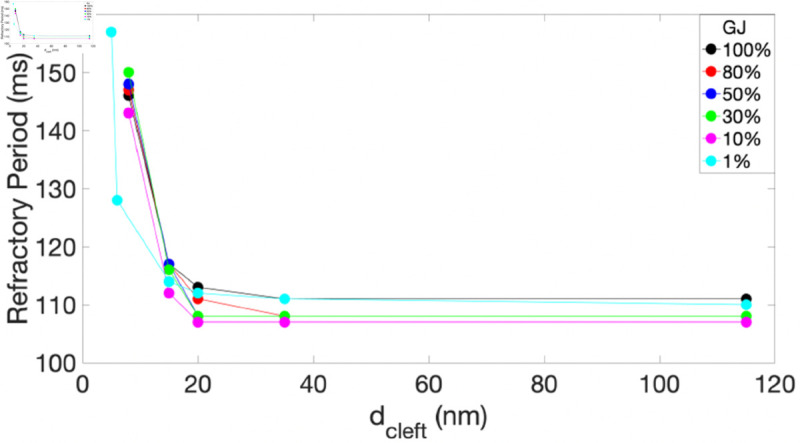
Rrefractory period as a function of dcleft, shown for different levels of GJs: 100% (black), 80% (red), 50% (blue), 30% (green), and 10% (magenta), 1% (cyan).

Two main mechanisms contribute to the suppression of reentry initiation, as demonstrated in our numerical simulations: (1) a prolonged refractory period and (2) an elevated anisotropy ratio with decreasing $d_{\text{cleft}}$. These effects are consistently observed across various levels of GJ coupling. Specifically, when EpC is strong—that is, when the cleft is sufficiently narrow (at $d_{\text{cleft}}=8$ nm or less)—the refractory period is markedly prolonged, leading to conduction delays or even CB, as illustrated in Fig 8, where the refractory period is shown as a function of $d_{\text{cleft}}$. This prolonged refractory period affects the formation and timing of reentry, potentially disrupting or even blocking the circular conduction pathway necessary for sustained reentry. In addition, the elevated anisotropy ratio observed at narrow clefts (Fig 5, right panel) also contributes to the inhibition of reentry initiation. Specifically, at $d_{\text{cleft}}=15$ nm and 20 nm, reentrant circuits may still initiate depolarization; however, when they encounter tissue that is already activated and refractory, the activity tends to propagate preferentially in the faster longitudinal direction. Meanwhile, transverse propagation is delayed and less effective, ultimately causing the reentrant wave to dissipate. Numerical simulations alone are not sufficient to fully uncover the mechanisms by which EpC influences reentry initiation, termination, and the resulting dynamics. To address this, we are currently applying dynamical systems analysis to a simplified model, aiming to identify the underlying mechanisms through the study of spiral wave dynamics.

The mathematical model employed in this study possesses several limitations. Specifically, our model overlooks intricate details such as the precise cell geometry, including any offsets, as well as the microdomain effects within the extracellular space and the intricate geometry of the intercalated discs. While these factors could be crucial for understanding reentry dynamics, incorporating them all would substantially escalate the computational demands of our large-scale 2D simulation. Consequently, this poses a significant challenge to our numerical investigations. However, addressing these aspects remains a focus of our future endeavors.

## Conclusion

The goal of this study is to explore how different levels of EpC and GJs influence the initiation and dynamics of reentry, utilizing a 2D discrete bidomain model of EpC. Specifically, we quantitatively assessed the incidence of reentry initiation and analyzed the subsequent dynamics across different levels of EpC, GJs, and initial perturbations. The results show that sufficiently strong EpC (i.e., sufficiently narrow clefts) tends to suppress reentry initiation, resulting in absent or non-sustained reentrant activity, while also introducing transient instability and heterogeneity into the cardiac dynamics. In contrast, relatively weak EpC (wide clefts) supports sustained reentry with a stable rotor. Furthermore, we found that sufficiently strong EpC can lower the maxDF observed during reentrant activity. Overall, this suggests that strong EpC exerts an anti-arrhythmic effect.

## Supporting information

The coordinates of points A, B, C, and D used in Figs 1 and 2 are provided below. These points are selected from the lattice model, where each coordinate is expressed as (row, column).

- For Fig 1 , the positions of the points are: A = (100, 100), B = (100, 400), C = (435, 260), and D = (300, 350).
- For Fig 2 , the positions of the points are: A = (387, 234), B = (100, 200), C = (414, 239), and D = (100, 400).
